# Physiology of Respiration and Chemistry of the Blood Applied to Epidemic Cholera

**Published:** 1835

**Authors:** Benjamin F. Joslin

**Affiliations:** Professor of Natural Philosophy, in Union College, N. Y.


VIII. *The Physiology of Respiration and Chemistry of the blood applied to Epidemic Cholera.* By Benjamin F. Joslin, M. D., Professor of Natural Philosophy, in Union College, N. Y. [Extracted from the Transactions of the Medical Society, of the state of New York.]

Dr. Joslin's essay contains the history of a series of original experiments on the influence of different agents on the color of the blood, which we propose to present to our readers in the following extract:—

*Experiments on the Blood.*—Great importance being generally attached to the deficiency of salts in cholera blood, and the following experiments, on blood nearly or exactly normal, differing perhaps in some respects, from those made by others, they may be worth recording. When disease existed it was slight, especially as to its effect on the blood. To prevent mistake it is necessary to state, that in all these experiments, I describe the color of the blood as seen by reflected light.

Venous blood was drawn from the arm of a man who had slight symptoms of fever, with gastro-intestinal irritation. The blood coagulated promptly and completely. The clot, as is usual, was dark colored beneath the surface. This was reddened by applying to it carbonate of soda in powder. On laying a lump of it on a dark portion of the coagulum, the surface in contact with the solid alkaline carbonate, though covered by it, was rendered of a scarlet color much more rapidly, than another portion wet with a saturated solution, and exposed as freely as possible to the air. The liquid which drained from the coagulum to which the dry soda was applied, as also the solution applied, was almost instantly rendered of a bright red color, not only at the surface, but throughout the interior, where no air was present, except the small quantity contained in the blood and in the solution. These experiments led me to suspect, that this alkaline salt has the property of changing the color of *any* dark venous blood to scarlet, and that less air is requisite than is generally believed.

The blood on which the next experiments were made, was drawn from one of the large veins of a hog, and received directly into four one ounce phials, which were completely filled. Three were instantly corked, and one left open. Of two of these which were corked, I had, before receiving the blood into them, put two scruples of carbonate of soda into each. One of these was then uncorked and immediately put under the receiver of an air pump, which was kept exhausted for ten or fifteen minutes, during which time considerable gas escaped from it. On removing it from the receiver, about one drachm of water was added, to compensate for the gas and blood which had escaped in the form of froth, and thus to exclude atmospheric air. When filled, it was immediately corked, and all the corked phials shortly after sealed, and kept at a temperature of about 60 deg. Fah. The color of both portions into which the soda had been put, was changed to scarlet, at the inferior part which was in contact with the salt or near it; but that from which the gases had not been removed, was much brighter than the other, and its coagulum had much less consistence. The two others without soda were sensibly alike in color, except that the one exposed to the air was reddened at the surface. The blood reddened by the soda, began, after an hour or two, to become sensibly darker, but after three hours, was still much brighter than the other, and that from which the gases had not been exhausted, was but little brighter, at that time, than that which had not been placed under the receiver. The next day, the order of brightness of the blood of the three closed phials, was completely reversed.

In the next series of experiments, a portion of florid blood drawn from the epigastrium of a man affected with slight chronic gastro-enteritis, was put into hot water. The coagula were blackened by it as usual. It has been asserted, that in such cases, the water extracts those salts on which the florid color depends. The following experiments proved, that the change of color is produced in such cases, neither by the extraction of salts nor by the extrication of oxygen. Half an ounce of the same blood in its florid state, was put with an equal quantity of water, into an ounce phial and boiled in a water bath. Both the liquid and solid parts became of a light olive color and a gaseous substance escaped. To determine the effect of a lower temperature, and to remove the ambiguity occasioned by the escape of the gases, as well as to determine the combined effect of heat and an alkaline salt on serum and red globules when separated from the fibrine of the crassamentum, two ounce phials were filled with the serous and aqueous liquid, in which the clot had been blackened, and to which it had imparted a florid color. A scruple of carbonate of soda was put into one of them, and both were then corked and immersed in a water bath, which was gradually heated to 150 degrees. At this temperature both became opaque; that which contained the soda soon becoming perfectly black as seen by reflected light, the other a dull brownish red. A half ounce phial was next filled with the same florid liquid, corked, sealed and immersed in water at 150 deg. Fah. It soon became opaque and brownish. A scruple of carbonate of soda was then added, which immediately rendered it black. Another portion was blackened by dilute nitric acid without heat. The blackness produced by the carbonate of soda remained after filtering through paper and exposure to air. A month afterwards, the black color remained unchanged, and was not affected by reheating the liquid to the temperature formerly employed. It is a curious fact established by the above experiments, that carbonate of soda produces the same black color in sanguineous coloring matter diffused through diluted serum and heated to 150 degrees Fah., that nitric acid does at 60 degrees. As a change of temperature is thus shown to modify and at length to reverse the effect produced by the salt, it is not improbable, that a repetition of the experiment with different salts and at different temperatures, might lead to interesting discoveries and suggest improvements in venous injection. It appears from these experiments, that the effect of saline substances on blood at one temperature, cannot be inferred from experiments made at another. These experiments moreover prove that the blackening of crassamentum by hot water is not dependent on the extraction of its saline matters; and also, that the change of color produced in sanguineous coloring matter by heat is not the result of the extrication of oxygen or any other gas. Indeed this change of color was produced by heat, when the phials were filled and perfectly closed, and was more remarkable when the saline matter exceeded the normal quantity.

Some of the preceding experiments were made (as has been stated) on serum and coloring matter only. In all the following experiments all the parts of the blood were in the same proportion as before its extraction from the vessels. Two one ounce phials full of venous blood, after having stood corked and sealed exactly six months, the one without any thing added, and the other with five grains of carbonate of potash, were heated in a water bath. At and above 140 degrees, considerable gas was evolved, but no change of color was produced by heating them to 155 degrees; the seals being then broken by the force of the gas and vapor, the experiment was discontinued. In the next experiment, five grains of carbonate of soda were put into a phial with ¼ ounce of blood extracted by cupping. Heated to 142 degrees it was a little darkened and at 150 degrees became black. A small portion seen by transmitted light appeared of a dark yellowish green color. The coagula appeared to have been rendered somewhat fluid. On raising the temperature, little change of color or consistence was observed till it reached 170 degrees, when the whole was found to have lost its fluidity and to have assumed the consistence of currant jelly. This consistence was not again diminished by cooling, but on the contrary increased. A heat of 150 degrees had rendered it black, whilst a heat of 170 degrees had coagulated the whole, without however giving it the tenacity or color of the original coagula. Even when cooled it had little of the elasticity of crassamentum, though it had no fluidity. No gas was evolved during any part of the process. The next and last experiment was made with three one ounce phials full of blood, which may be distinguished as A, B and C. A, was venous and arterial blood obtained by cupping, and simply sealed. B and C venous blood, the two portions exactly alike. To B, grs. v. carbonate of soda were added; to C, grs. v. of the carbonate of the same alkali. All being sealed were exposed to the same heat.—The following were the results: The color of A, was not sensibly changed till heated to about 170 degrees, when it underwent a kind of coagulation, assuming a consistence and elasticity like that of a solution of glue when cold, its color simultaneously changing to an olive grey. B became black at 150 degrees, (its color beginning to change at about 148 degrees.) This blackness extended only as far as the previous reddening influence of the carb. sod., the rest remaining of the original dark red color. C was not in the least darkened at 150 degrees; but where the bicarbonate had come in contact with it, it still retained that excess of brightness which the salt had communicated, till heated to some point between 160 degrees and 170 degrees, when it became dark, but not as black as B. From a comparison of B and C, I infer that that salt of soda, which contains most carbonic acid requires a higher temperature to render blood dark, and blackens it but imperfectly, even at a higher temperature. From these last experiments, and from a comparison of those on A, with similar ones which have been described above, and which were made on the same kind of blood, I infer that the carbonate of soda with heat, blackens both venous and arterial blood. The coagulation of the mixed blood at 170 degrees was effected by heat alone. These last experiments then, though made under circumstances which developed some new and curious phenomena, tended to confirm and render more general the preceding comclusions in regard to the blood."

After this statement of his experiments, our author proceeds to insist, on a close analogy between the state of the blood in Epidemic Cholera and asphyxia, in both of which, he believes, it is loaded with carbon, and has its coagulability much diminished, or entirely destroyed. He does not attach much importance to the loss of serum, and is from principle altogether sceptical as to the beneficial influence of saline injections into the veins. Of the manner in which this highly carbonaceous condition of the blood is brought about he does not pretend to be satisfied, but presumes the remote cause to exert itself upon the lungs. The prominent symptoms and fatal termination of the disease may, he thinks be fairly attributed to this altered condition of the blood, disturbing and prostrating the different functions, as they are annihilated in asphyxia.

Under the head *aetiology*, our author recognizes the existence of an unknown ærial cause as predisposing to the disease, and the following auxiliary or exciting causes.

"1st, Heat; 2d, Long-continued and violent exercise; 3d, Alcohol; 4th, Fasting; 5th, The depressing passions; 6th, Night; 7th, Vegetable diet. To these should probably be added—Cutaneous Filth."

The whole of these influences, he supposes, co-operate with the unknown agent in diminishing the oxygenation or decarbonization of the blood, and subjoins to each an argument to show that its effect is of that kind.

The *modus agendi* of this undiscovered cause may be such as to lead to the same state of the blood, carried to a higher degree than the common causes assigned by Dr. Joslin could have produced; or it may have acted in some other mode, so as to give to defective oxygenation from the conditions he has assigned, a noxious influence greater, than it would otherwise have effected. However, this may be, we perceive the necessity of assuming the existence of such a cause, and of placing it in the rank of a principal and predisposing influence. It is far from our intention to speculate on the nature of this cause, but, of course, we believe it to be connected with the atmosphere; and it may, perhaps, consist in one of the following conditions—first a diminution in the proportion of oxygen; a defect insisted upon by us in the last number of this journal; but which, it must be confessed, is only a hypothesis;—second a modification of the electro — magnetic state of the air — likewise hypothetical;—third, the presence in the air of some noxious agent. In adopting either of these suppositions, we are, however, met, in a palpable manner, by the fact, that Epidemic Cholera is a progressive disease—that it traveled from India, within the tropics to Russia where it spread almost at the arctic circle; that it passed over to Great Britain, thence to Canada, thence to the shores of the Hudson, and the Ohio; that advancing rapibly to Louisiana, it extended round the Gulf of Mexico, and soon after invaded South America and the West Indies; that from the north and east of Europe it descended upon the south-western kingdoms of that continent, and still lingers on the shores of the Mediterranean. To such a progressive spread among the nations of the earth we consider it impossible at present to reconcile any of the theories we have cited.

Our limits do not permit us to give as full an analysis of Dr. Joslin's paper as its merits deserve. We have not for some time looked over a paper on the physiology and pathology of cholera, that was written in such a philosophical spirit, and presented as many sound views of the subject; or as sensible an application of the science of chemistry to that of medicine. We hope the Medical Society of thestate of New-York will long continue to favor the profession with such papers.

